# Clinical characteristics and real-world survival in acral melanoma: experience from a comprehensive cancer center in Latin America

**DOI:** 10.3389/fonc.2026.1828547

**Published:** 2026-05-13

**Authors:** Diego Gómez-Abreo, Michael Steven Villabona Díaz, Nathalia Andrea Olarte-Licht, Lady Katerine Aldana Galvis, Johan Danilo Torres Rosas, Alexandra Hurtado-Ortiz, Maricel Licht-Ardila, Edgar Fabián Manrique-Hernández

**Affiliations:** 1 Department of Oncology, Hospital Internacional de Colombia (HIC), Fundación Cardiovascular de Colombia (FCV), Floridablanca, Santander, Colombia; 2 Department of Epidemiology, Hospital Internacional de Colombia (HIC), Fundación Cardiovascular de Colombia (FCV), Floridablanca, Santander, Colombia

**Keywords:** Colombia, developing countries, melanoma, prognosis, survival analysis

## Abstract

**Background:**

Acral melanoma represents a biologically distinct and clinically aggressive melanoma subtype disproportionately affecting non-Caucasian populations. Evidence from low- and middle-income countries remains limited, and the real-world impact of modern immunotherapy in these patients is not well characterized.

**Objective:**

To describe clinical characteristics, treatment patterns, and survival outcomes of patients with acral melanoma treated at a comprehensive cancer center in Latin America.

**Methods:**

We conducted a retrospective cohort study including adult patients with histologically confirmed acral melanoma involving the palms, soles, or nail apparatus. Sociodemographic, clinical, pathological, and treatment data were collected. Overall survival was estimated using the Kaplan–Meier method and compared with the log-rank test.

**Results:**

Forty-seven patients with acral melanoma were included; 18 (38.3%) died during follow-up. Median age at diagnosis was 65.3 years, with less favorable survival observed among older patients. Estimated overall survival was 62.2% at 1 year and 59.4% at both 3 and 5 years. Females and patients aged ≤65 years showed higher survival estimates across follow-up. Immunotherapy was the most frequently used systemic treatment.

**Conclusions:**

Acral melanoma in this Latin American cohort showed unfavorable long-term survival, consistent with late-stage presentation and potential delays in detection, highlighting ongoing diagnostic and treatment shortfalls in middle-income settings. Real-world experience suggests that immunotherapy is being adopted in routine practice, while access to specialized care remains uneven. Multicenter regional studies are needed to better characterize outcomes and inform context-appropriate management strategies in underrepresented populations.

## Introduction

Cutaneous melanoma is an aggressive malignant neoplasm arising from melanocytes and represents one of the fastest-growing cancer incidences worldwide, constituting a significant public health burden due to its metastatic potential and high disease-specific mortality (1, 2). While most melanoma subtypes are strongly associated with intermittent or cumulative ultraviolet radiation (UVR) exposure, acral melanoma (AM), arising on the palms, soles, and nail apparatus, is considered a biologically and epidemiologically distinct entity (3, 4).

Biologically, AM displays markedly lower UVR-induced mutational signatures and exhibits a predominance of structural genomic alterations, including copy number variations, chromosomal rearrangements, and amplifications involving oncogenic pathways, supporting a UV-independent carcinogenic mechanism (5, 6). This molecular divergence contributes to lower tumor mutational burden and differential patterns of targetable alterations compared with other cutaneous melanomas (5).

Epidemiologically, AM accounts for only 2–3% of melanoma cases in predominantly European-descendant populations but represents a substantially higher proportion, and in some regions the most prevalent melanoma subtype, among individuals of African, Asian, and Hispanic ancestry (4, 7). This ethnic and geographical distribution underscores persistent disparities in diagnosis, treatment, and survival, reflecting both biological variability and contextual determinants of health.

Historically, AM has been associated with poorer outcomes relative to non-acral melanoma. Though biological aggressiveness has been proposed, delayed diagnosis, greater Breslow thickness at presentation, ulceration, and sentinel lymph node involvement are consistently identified as major contributors to the survival gap (8, 9). These factors are particularly consequential in low- and middle-income countries (LMICs), where barriers such as limited access to specialized dermatologic care, delayed referral pathways, and restricted availability of advanced diagnostic and systemic therapies persist (10–12). In these settings, AM is frequently diagnosed at advanced stages, leading to inferior survival and mortality profiles compared with cohorts reported from high-income countries.

Although evidence from LMICs remains limited, some regional cohorts have begun to characterize acral melanoma outcomes in underrepresented populations. Studies from Latin America, including cohorts from Colombia and Mexico, have reported advanced stage at diagnosis and heterogeneous survival outcomes, highlighting persistent diagnostic delays and health system constraints (13, 14). Similarly, data from Asian and African populations have shown distinct clinicopathological patterns and variable prognosis compared with high-income settings (15, 16). However, these studies remain limited in size, setting, and access to modern systemic therapies, restricting their generalizability and limiting their ability to fully capture real-world treatment patterns and outcomes.

Despite this disproportionate burden, evidence regarding clinical characteristics, treatment patterns, and survival determinants of AM in LMICs remains scarce. The vast majority of existing data originates from high-income Asian or North American cohorts, limiting generalizability to Latin American populations, where genetic ancestry, health system structure, and therapeutic access diverge substantially (9). Therefore, this study aims to describe clinical characteristics, treatment patterns, and survival outcomes of patients with acral melanoma treated at a comprehensive cancer center in Latin America.

## Methods

### Study design and setting

We conducted a retrospective cohort study at a tertiary cancer center in Colombia. The institution provides specialized oncologic care, onsite dermatopathology, and access to systemic therapies including immunotherapy, making it a representative setting for real-world melanoma care in a middle-income country context.

### Study population

Eligible patients were adults aged ≥18 years with a histologically confirmed diagnosis of AM, defined as melanoma arising on the palms, soles, or nail apparatus. Cases between July 2017 and December 2025 were identified through linked institutional clinical, administrative, and pathology databases using diagnostic codes and oncologist-confirmed records. Only patients with a documented diagnosis date and at least one consultation with an oncology specialist were included.

### Inclusion and exclusion criteria

We included patients with: (1) histologically confirmed acral melanoma, (2) available baseline clinical and pathological data, and (3) at least one follow-up record after diagnosis allowing outcome assessment. We excluded patients with melanomas arising at non-acral sites, those with recurrent disease diagnosed prior to the study period, and cases with missing key variables required for survival analysis (including date of diagnosis, vital status, or follow-up information). Patients lost to follow-up immediately after diagnosis without outcome information were also excluded from survival analyses.

### Data sources and variables

Data were obtained from electronic health records and institutional databases using unique patient identifiers. Variables collected included: Sociodemographic variables: sex, age at diagnosis, residence (urban vs. rural), educational level and marital status. Clinical variables: primary tumor site (palm, sole, nail apparatus, mixed, acral unspecified), date of diagnosis, and clinical stage at diagnosis. Disease staging followed the 8th edition of the American Joint Committee on Cancer (AJCC) TNM system (17). The acral score was defined as the number of acral subregions involved at diagnosis (range 1–5), including plantar surface, palmar surface, nail apparatus, heel, and digits. Histopathological variables: Breslow thickness (18), ulceration, mitotic rate, and other pertinent pathological features. When multiple specimens were available (e.g., biopsy and wide excision), the highest-risk pathological characteristic was recorded, consistent with prior melanoma cohort methodology (19, 20).

Treatment-related variables: initial management modality (surgery, radiotherapy, systemic therapy, palliative care, or no treatment), type of systemic therapy (including immune checkpoint inhibitors), number of treatment lines, and best documented treatment response.

### Outcomes and definitions

Disease progression, site(s) of progression, metastatic involvement, last follow-up date, and vital status were systematically recorded. Overall survival (OS) was defined as the time from histologic diagnosis to death from any cause or last documented follow-up. Progression-free survival (PFS) was not evaluated due to limitations in the availability and consistency of progression data in this retrospective cohort.

### Statistical analysis

Descriptive statistics were used to summarize cohort characteristics, treatment patterns, and outcomes. Categorical variables were reported as frequencies and percentages. Continuous variables were summarized as median (interquartile range), according to distribution assessed using the Shapiro–Wilk test. Overall survival (OS) was estimated using the Kaplan–Meier method, and differences between groups were evaluated using the log-rank test. Bivariate analyses were conducted to explore associations between sociodemographic, clinical, pathological, and treatment-related variables and survival outcomes. Categorical variables were compared using the chi-square test or Fisher’s exact test, and continuous variables using the Mann–Whitney U test, as appropriate.

Missing data were handled using a complete-case approach for inferential analyses. Variables with a high proportion of missing data (>40%) were primarily interpreted descriptively. No imputation procedures were performed. Results derived from these variables should therefore be interpreted with caution as exploratory findings. Given the limited sample size and number of outcome events, multivariable modeling was not performed to avoid model overfitting and unstable estimates. All analyses were performed using Stata version 16 (StataCorp LLC, College Station, TX, USA), with a two-sided significance level of p < 0.05.

#### Ethical considerations

The study was conducted in accordance with the Declaration of Helsinki and Colombian national regulations governing research involving human subjects. Given its retrospective design and use of secondary clinical data, it was classified as minimal risk. Institutional ethics approval was obtained (CEI-2024-07461-32), and data confidentiality was ensured through anonymization and restricted-access protocols.

## Results

The cohort included 47 patients with AM. At the end of follow-up, 29 patients (61.7%) were alive and 18 (38.3%) had died. Patients who died were older at diagnosis, with a median age of 78.5 years compared with 60.7 years among survivors. This difference was statistically significant (p = 0.018), underscoring the relevance of age as a key demographic characteristic within the cohort. (**Table 1**). Tumor distribution across plantar, and mixed acral sites, as well as the acral location score (median 3), was similar between groups, supporting the comparability of baseline clinicopathological characteristics between survivors and non-survivors.

**Table 1:** Sociodemographic characteristics of patients with acral melanoma stratified by survival status.

Variable	Alive *n* (%)	Dead *n* (%)	Total *n* (%)	*p*-value
Sex				0.120
Female	21 (72.4)	9 (50.0)	30 (63.8)	
Male	8 (27.6)	9 (50.0)	17 (36.2)	
Age (years)	60.7 (56.6–68.4)	78.5 (57.4–85.7)	65.3 (56.7–79.7)	0.018
Educational level				0.583
No formal education	1 (3.5)	0 (0.0)	1 (2.1)	
Primary education	18 (62.1)	8 (44.4)	26 (55.3)	
Secondary/Technical	5 (17.2)	6 (33.3)	11 (23.4)	
Higher education	2 (6.9)	1 (5.6)	3 (6.4)	
Not applicable/Missing	3 (10.3)	3 (16.7)	6 (12.8)	
Marital status				0.969
Married/Cohabiting	15 (51.7)	9 (50.0)	24 (51.1)	
Single/Separated/Widowed	12 (41.4)	8 (44.4)	20 (42.6)	
Not reported	2 (6.9)	1 (5.6)	3 (6.4)	
Area of residence				0.236
Rural	4 (13.8)	5 (27.8)	9 (19.1)	
Urban	25 (86.2)	13 (72.2)	38 (80.9)	

Data are presented as *n* (%) or median (interquartile range). Comparisons were performed using χ² or Fisher’s exact test for categorical variables and the Mann–Whitney U test for continuous variables. *p* < 0.05 was considered statistically significant.

Differences emerged in disease burden and evolution. Advanced disease at diagnosis (stages III–IV) was more frequent among deceased patients, whereas earlier stages predominated among survivors. Metastatic disease was documented in 18 patients (38.3%) and was more frequent among those who died (12/18, 66.7%) than among survivors (6/29, 20.7%); however, metastatic status was not available for 23 patients (48.9%). Disease progression was also more common among patients who died (55.6%) than among survivors (24.1%). Initial treatment was predominantly surgical in both groups (**Table 2**).

**Table 2:** Clinical, pathological, and treatment-related characteristics of patients with acral melanoma stratified by survival status.

Variable	Alive *n* (%)	Dead *n* (%)	Total *n* (%)	*p*-value
**Acral score**	3 (2–5)	3 (2–4)	3 (2–5)	0.901
Anatomical region				0.441
Plantar	9 (31.0)	6 (33.3)	15 (31.9)	
Palmar	2 (6.9)	3 (16.7)	5 (10.6)	
Ungual	2 (6.9)	2 (11.1)	4 (8.5)	
Mixed	12 (41.4)	7 (38.9)	19 (40.4)	
Acral unspecified	4 (13.8)	–	4 (8.5)	
Stage (AJCC)				0.073
In situ	1 (3.5)	–	1 (2.1)	
Stage I	4 (13.8)	–	4 (8.5)	
Stage II	9 (31.0)	2 (11.1)	11 (23.4)	
Stage III	8 (27.6)	7 (38.9)	15 (31.9)	
Stage IV	2 (6.9)	6 (33.3)	8 (17.0)	
Not evaluated	5 (17.2)	3 (16.7)	8 (17.0)	
Initial treatment				0.264
Surgical management	26 (89.7)	13 (72.2)	39 (83.0)	
No treatment	3 (10.3)	3 (16.7)	6 (12.8)	
Radiotherapy	–	1 (5.6)	1 (2.1)	
Palliative care	–	1 (5.6)	1 (2.1)	
Metastasis				0.014
No metastasis	6 (20.7)	–	6 (12.8)	
Metastasis present	6 (20.7)	12 (66.7)	18 (38.3)	
No information	17 (58.6)	6 (33.3)	23 (48.9)	
Progression				0.040
No progression	5 (17.2)	–	5 (10.6)	
Progression	7 (24.1)	10 (55.6)	17 (36.2)	
Not evaluated	17 (58.6)	8 (44.4)	25 (53.2)	

Data are presented as *n* (%) or median (interquartile range). Comparisons were performed using χ² or Fisher’s exact test for categorical variables and the Mann–Whitney U test for continuous variables. Tumor staging followed the AJCC TNM classification (8th edition). *p* < 0.05 was considered statistically significant. p-values were calculated using complete-case analysis (excluding missing data).

### Survival

**Figure 1:**
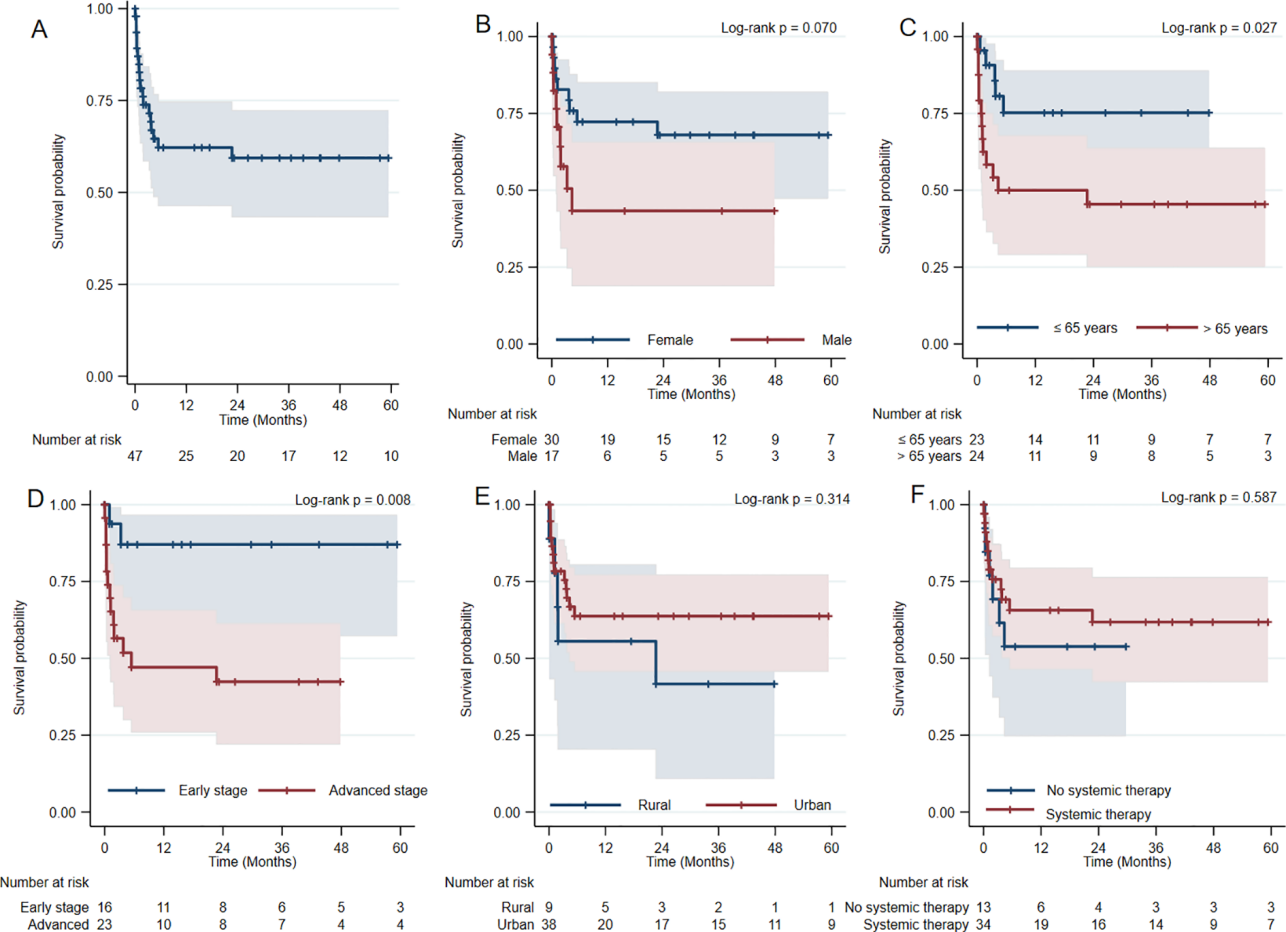
Kaplan–Meier overall survival curves stratified by sociodemographic, clinical, and treatment-related variables in patients with acral melanoma. **(A)** Overall cohort. **(B)** By sex. **(C)** By age group (≤65 vs. >65 years). **(D)** By disease stage (early vs. advanced). **(E)** By area of residence. **(F)** By systemic therapy. Shaded areas indicate 95% confidence intervals; log-rank tests were used for comparisons.

The median follow-up time was 15.6 months. OS declined progressively during follow-up, with most deaths occurring within the first years after diagnosis. The estimated 1-year OS was 62.20% (95% CI: 46.35 – 74.59), decreasing to 59.4% (95% CI: 43.3–72.3) in 3 years. The 5-year OS remained at 59.4% (95% CI: 43.3–72.3). Kaplan–Meier estimates showed an early decline in survival followed by a plateau, indicating that mortality events were concentrated in the initial phase after diagnosis (**Figure 1A**).

When stratified by sex, females showed higher survival probabilities across follow-up. Estimated OS among females was 72.3% (95% CI: 52.1-85.1) at 1 year and 68.0% (95% CI: 47.3-82.0) at both 3 and 5 years. Among males, estimated OS was 43.3% (95% CI: 18.9–65.7) at 1, 3 and 5 years (**Figure 1B**).

Clear differences in survival patterns were observed according to age at diagnosis. Patients aged ≤65 years showed an estimated OS of 75.2% (95% CI: 50.2-88.9) at 1, 3 and 5 years. In contrast, patients older than 65 years exhibited lower survival probabilities, with estimated OS of 50.0% (95% CI: 29.1-67.8) at 1 year, 45.5% (95% CI: 25.2-63.7) at 3 and 5 years (**Figure 1C**).

Survival also varied according to disease stage at diagnosis. Patients with early-stage melanoma (stages 0–II) showed an estimated OS of 87.1% (95% CI: 57.3-96.6) at 1, 3 and 5 years. In contrast, patients diagnosed with advanced-stage disease (stages III–IV) had lower survival probabilities, with estimated OS of 47.1% (95% CI: 26.0-65.7) at 1 year, 42.4% (95% CI: 22.1–61.4) at 3 and 5 years (**Figure 1D**).

According to area of residence, patients from rural areas showed an estimated OS of 55.6% (95% CI:20.4-80.5) at 1 and 5 years. Urban residents had an estimated OS of 63.7% (95% CI: 45.8–77.1) at 1, 3 and 5 years (**Figure 1E**).

Patients who did not receive systemic therapy showed an estimated OS of 53.8% (95% CI: 24.8-76.0) at 1 and 5 years. Among patients who receive systemic therapy, estimated OS was 65.7% (95% CI: 46.5-79.4) at 1 year, 61.8% (95% CI: 42.4-76.4) at 3 and 5 years (**Figure 1F**).

### Systemic therapy

Among patients with available data, 19 received first-line systemic therapy, 7 second-line therapy, and 3 third-line therapy. Immune checkpoint inhibitor–based regimens were the most frequently used across all treatment lines. Systemic therapy was administered according to disease stage and clinical indication, including adjuvant treatment for selected patients with high-risk stage III disease and systemic treatment for unresectable or metastatic stage IV melanoma. In first-line therapy, complete response was observed in 6 patients (31.6%), disease progression in 5 (26.3%), and 7 patients (36.8%) were not evaluable at the time of assessment. In the second-line setting, disease progression was the most common outcome, occurring in 3 of 7 patients (42.9%), while complete or partial responses were observed in 2 patients (28.6%). Among the 3 patients receiving third-line therapy, one achieved complete response, one experienced disease progression, and one was not evaluable. Overall, immunotherapy predominated as the systemic strategy in this cohort, with heterogeneous response patterns observed across therapy lines (**Table 3**).

**Table 3:** Response to treatment according to therapy line and treatment modality.

Line	Response	Immunotherapy	Interferon/Chemotherapy	Radiotherapy	Targeted therapy	Total	*p*-value
1L (n=19)	CR	5 (33.3%)	1 (50.0%)	-	-	6 (31.6%)	0.648
	NE	6 (40.0%)	–	1 (100%)	–	7 (36.8%)	
	PD	3 (20.0%)	1 (50.0%)	–	1 (100%)	5 (26.3%)	
	SD	1 (6.7%)	–	–	–	1 (5.3%)	
2L (n=7)	CR	1 (20.0%)	–	–	–	1 (14.3%)	0.857
	NE	–	1 (100%)	–	–	1 (14.3%)	
	PD	2 (40.0%)	–	–	1 (100%)	3 (42.9%)	
	PR	1 (20.0%)	–	–	–	1 (14.3%)	
	SD	1 (20.0%)	–	–	–	1 (14.3%)	
3L (n=3)	CR	1 (100%)	–	–	–	1 (33.3%)	1.000
	NE	1 (100%)	–	–	–	1 (33.3%)	
	PD	–	–	–	1 (100%)	1 (33.3%)	

1L, first-line therapy; 2L, second-line therapy; 3L, third-line therapy; CR, complete response; PR, partial response; SD, stable disease; PD, progressive disease; NE, not evaluable.

## Discussion

In this real-world cohort from a tertiary cancer center in Colombia, overall survival in patients with AM appeared to vary mainly according to age and stage at diagnosis. These observations add region-specific evidence from a middle-income country (MIC) and underscore the combined influence of tumor biology and health system–related factors in shaping outcomes for this uncommon and clinically challenging melanoma subtype.

The 5-year overall survival observed in our cohort was comparable to estimates reported in high-income settings, where survival probabilities typically range between 50% and 70%, depending on stage distribution, access to therapies, and use of immunotherapy (4, 21). This similarity suggests that geographic location alone does not fully explain survival differences in acral melanoma. Rather, intrinsic biological features, diagnostic delays, and heterogeneity in treatment pathways likely play a central role. Notably, a substantial proportion of patients in our cohort were diagnosed at advanced stages, consistent with previous reports indicating that acral lesions often arise in non–sun-exposed and visually concealed sites, which may delay clinical recognition and referral (22). Importantly, our findings are also consistent with previously reported cohorts from LMICs, including studies from Mexico, Colombia, China, and South Africa (13–16), where late-stage presentation and unfavorable survival patterns have been similarly described, reinforcing the role of systemic and access-related barriers across diverse settings.

Older age at diagnosis was associated with less favorable survival patterns in this cohort. This finding aligns with population-based and institutional studies showing that advanced age is linked to poorer melanoma outcomes (23, 24). Beyond comorbidity burden and treatment eligibility, age-related changes in immune function may also influence outcomes, particularly in acral melanoma, which is characterized by lower immunogenicity compared with other cutaneous melanoma subtypes (25).

Stage at diagnosis remained a key factor in survival trajectories, with patients presenting with early-stage disease showing more favorable outcomes than those diagnosed at advanced stages. This pattern mirrors global experience and reinforces the central role of early detection in melanoma prognosis (17). In MIC settings, this challenge may be amplified by structural barriers such as limited access to dermatologic expertise, lower awareness of acral melanoma among patients and providers, and fragmented referral pathways, all of which may contribute to delayed diagnosis (26). Similar diagnostic delays and stage distributions have been consistently reported in LMIC cohorts, supporting the need for improved early detection strategies and strengthening of referral systems in resource-constrained settings (13–16).

Differences in survival according to sex and area of residence were less pronounced in this cohort. Although male sex has been associated with poorer melanoma outcomes in several large studies (27), only modest differences were observed here. Similarly, while rural residence has been linked to delayed melanoma diagnosis in both low- and high-income settings, the centralized care provided at a referral cancer center may have partially mitigated geographic disparities in this cohort. This observation may also reflect selection patterns inherent to tertiary referral centers, where access to specialized care could attenuate disparities typically observed at the population level.

Interpretation of systemic therapy outcomes warrants caution. The absence of clear survival differences according to receipt of systemic therapy likely reflects limited sample size, heterogeneity in treatment indications, and potential confounding by disease severity. Importantly, acral melanoma is known to harbor a distinct genomic landscape, characterized by low tumor mutational burden, UV-independent mutational signatures, and a predominance of structural alterations, including KIT mutations and amplifications of CCND1 and CDK4 (5, 6, 27). These features may reduce immunogenicity and contribute to variable responsiveness to immune checkpoint inhibitors, which depend on neoantigen-driven immune activation. Real-world studies have reported lower response rates and shorter progression-free survival with anti–PD-1 therapies in acral melanoma compared with non-acral cutaneous melanoma, although durable responses have been documented in selected patients (28, 29). Comparable variability in treatment response has also been described in LMIC cohorts, where differences in access to systemic therapies and delays in treatment initiation may further influence outcomes (13–16).

Despite these biological considerations, systemic treatment patterns in this cohort reflect contemporary real-world practice, with immune checkpoint blockade representing the most commonly used approach. The use of alternative systemic strategies in a subset of patients highlights treatment heterogeneity and access-related constraints, a scenario frequently encountered in MIC oncology settings (11). These findings emphasize the need to contextualize therapeutic outcomes within local health system capacities and resource availability.

The observed relation between metastatic progression and mortality further underscores the aggressive clinical course of acral melanoma once systemic dissemination occurs. This observation is consistent with prior studies identifying metastatic progression as a pivotal event in the disease trajectory and a major determinant of survival (30).

Some limitations should be acknowledged. The retrospective, single-center design may introduce selection bias and limit external validity, particularly given the referral patterns inherent to tertiary care institutions. The relatively small sample size reduces statistical power and may increase the risk of type II error, especially in subgroup analyses. In addition, a considerable proportion of missing data in key variables, such as metastatic status and disease progression, may have introduced information bias. Analyses involving these variables were therefore based on complete-case data and should be interpreted as exploratory.

Furthermore, overall survival was assessed using all-cause mortality, which does not allow attribution of death specifically to melanoma and may introduce confounding, particularly in older patients with competing risks of non-cancer mortality. Consequently, associations observed with survival, especially those related to age, should not be interpreted as melanoma-specific effects. Incomplete molecular profiling precluded detailed analyses of biomarker-driven treatment strategies. Additionally, progression-free survival was not evaluated due to inconsistent documentation of disease progression and heterogeneous follow-up intervals, which may limit the reliability of this endpoint in retrospective real-world settings.

Nevertheless, the study’s strengths include comprehensive clinical characterization, extended follow-up, and the generation of real-world evidence from Latin America, a region that remains underrepresented in the melanoma literature despite its diverse genetic ancestry and distinct health system context. The inclusion of patients treated in the era of immune checkpoint inhibitors and the use of standardized survival analysis methods further strengthen the robustness and contemporary relevance of the findings.

Overall, these findings suggest that improving outcomes in acral melanoma will require coordinated advances in early detection, molecular characterization, and health system performance. The biological distinctiveness of this subtype supports further investment in translational research and biomarker development, while the frequent presentation at advanced stages highlights persistent diagnostic and referral gaps. In MIC settings, strengthening provider education, improving referral networks, and expanding equitable access to specialized melanoma care and systemic therapies represent key opportunities to reduce outcome disparities. Multicenter and regional collaborations will be essential to advancing knowledge and optimizing care for this historically understudied melanoma subtype.

## Conclusion

Despite therapeutic advances, acral melanoma in this cohort remained associated with unfavorable survival outcomes, largely shaped by older age and advanced stage at diagnosis. Addressing diagnostic delays and expanding access to specialized melanoma care constitute the most immediate opportunities to improve outcomes for patients with acral melanoma in middle-income countries.

## Data Availability

The raw data supporting the conclusions of this article will be made available by the authors, without undue reservation.
